# Enhancement of herbicolin A production by integrated fermentation optimization and strain engineering in *Pantoea agglomerans* ZJU23

**DOI:** 10.1186/s12934-023-02051-z

**Published:** 2023-03-13

**Authors:** Hongkai Wang, Yaqi Zhou, Sunde Xu, Boyan Zhang, Tomislav Cernava, Zhonghua Ma, Yun Chen

**Affiliations:** 1 State Key Laboratory of Rice Biology, Key Laboratory of Molecular Biology of Crop Pathogens and Insects, Department of Plant Protection, Zhejiang University, Hangzhou, China; 2 Institute of Environmental Biotechnology, Graz University of Technology, Graz, Austria

**Keywords:** Herbicolin A, *P. agglomerans*, Fermentation optimization, Strain engineering, Antifungal activity

## Abstract

**Background:**

The lipopeptide herbicolin A (HA) secreted by the biocontrol agent *Pantoea agglomerans* ZJU23 is a promising antifungal drug to combat fungal pathogens by targeting lipid rafts, both in agricultural and clinical settings. Improvement of HA production would be of great significance in promoting its commercialization. This study aims to enhance the HA production in ZJU23 by combining fermentation optimization and strain engineering.

**Results:**

Based on the results in the single-factor experiments, corn steep liquor, temperature and initial pH were identified as the significant affecting factors by the Plackett–Burman design. The fermentation medium and conditions were further optimized using the Box-Behnken response surface method, and the HA production of the wild type strain ZJU23 was improved from ~ 87 mg/mL in King’s B medium to ~ 211 mg/mL in HA induction (HAI) medium. A transposon library was constructed in ZJU23 to screen for mutants with higher HA production, and two transcriptional repressors for HA biosynthesis, LrhA and PurR, were identified. Disruption of the *LrhA* gene led to increased mRNA expression of HA biosynthetic genes, and subsequently improved about twofold HA production. Finally, the HA production reached ~ 471 mg/mL in the Δ*LrhA* mutant under optimized fermentation conditions, which is about 5.4 times higher than before (~ 87 mg/mL). The bacterial suspension of the Δ*LrhA* mutant fermented in HAI medium significantly enhanced its biocontrol efficacy against gray mold disease and Fusarium crown rot of wheat, showing equivalent control efficacies as the chemical fungicides used in this study. Furthermore, HA was effective against fungicide resistant *Botrytis cinerea*. Increased HA production substantially improved the control efficacy against gray mold disease caused by a pyrimethanil resistant strain.

**Conclusions:**

This study reveals that the transcriptional repressor LrhA negatively regulates HA biosynthesis and the defined HAI medium is suitable for HA production. These findings provide an extended basis for large-scale production of HA and promote biofungicide development based on ZJU23 and HA in the future.

**Graphical Abstract:**

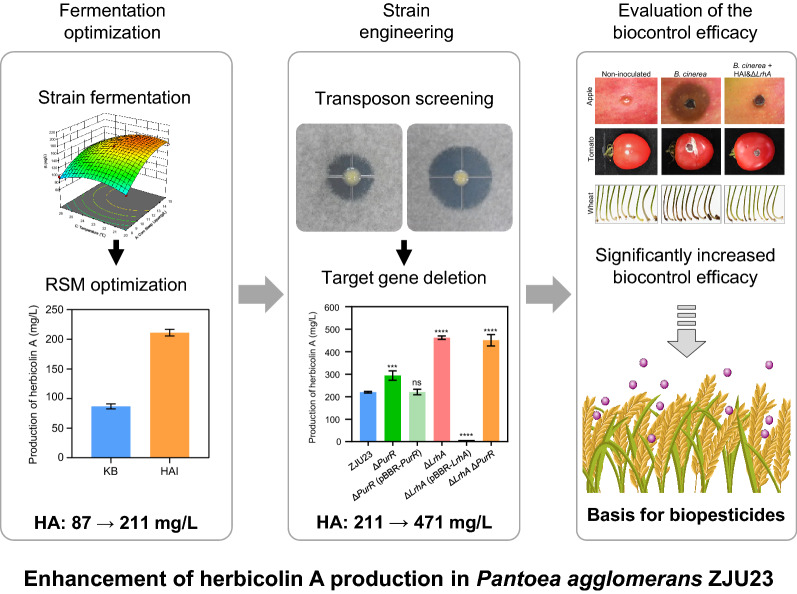

**Supplementary Information:**

The online version contains supplementary material available at 10.1186/s12934-023-02051-z

## Background

Food production needs to increase by 60% to meet the demands of approximately 10 billion people around the world by 2050 [1]. The fast-growing human population requires an increased crop yield, along with a reduction in crop loss caused by crop pathogens and pests. An expert-based assessment of crop losses on an individual pathogen and pest basis for wheat, rice, maize, potato and soybean, as five major crops, globally indicated that the average of crop losses caused by pathogens and pests are about 22.5% for all five crops [2]. Wheat is the staple crop for an estimated 35% of the world population [3]. A critical approach to meeting the increased demand is better management of fungal diseases, which can be responsible for 15%–20% yield losses per annum, such as rusts, blotches and Fusarium head blight (FHB) [4]. For example, the annual average occurrence of FHB, which is mainly caused by *Fusarium graminearum* in China, affects more than 4.5 million hectares, approximately 20% of the total planted area of wheat, and has caused serious yield losses [5]. Furthermore, FHB pathogens contaminate grains with various mycotoxins, especially deoxynivalenol (DON), which poses a health threat to humans and livestock. Currently, chemical fungicides are still the most effective approach to control FHB. However, fungicide resistant *F. graminearum* isolates have been detected in fields after long-term intensive use of fungicides [6, 7]. Moreover, treatments with some fungicides at sub-lethal concentrations stimulate mycotoxin production [8–10]. There is an urgent need to develop and apply new approaches to control FHB and mycotoxin contamination.

Biological control by living microorganisms is considered to be a suitable, alternative strategy for the control of plant diseases. Several antagonistic microorganisms were identified as biocontrol agents (BCAs) to combat plant diseases and achieved comparatively good efficiency [11]. Moreover, more than 30 species of microbes have been reported to specifically inhibit the mycelial growth of *F. graminearum*, or/and provided successful control efficiencies against FHB and mycotoxin reduction under greenhouse and/or field conditions [12–15]. Multiple mechanisms are involved in the biocontrol of plant diseases, including direct inhibition of pathogens by antimicrobial substances, competition, lysis and parasitism, or indirect interactions, e.g. induction of plant resistance [11, 16]. The most extensive studies on biocontrol mechanisms targeted the suppression of pathogens through antimicrobial substances. For example, bacterial BCAs can secrete lipopeptides, phenazine derivatives, macrolide antibiotics, nitrogenous heterocyclic compounds and other antifungal metabolites to directly inhibit *F. graminearum* [11, 15, 17, 18]. Among them, amphiphilic compounds, especially lipopeptides, have been extensively investigated [19]. More than 200 lipopeptide compounds are reported so far and can exhibit potent antimicrobial activities against various pathogens, both in agricultural and clinical settings [20]. For example, biocontrol agents belonging to *Bacillus* spp. were shown to secrete iturins, fengycins and polymyxins, which can suppress plant fungal diseases through various modes of action by interacting with fungal membranes, cell wall, or acting as nucleic acid inhibitors [21–25]. Eight lipopeptides have been developed as drugs to treat infections in clinics, such as daptomycin, micafungin and caspofungin (go.drugbank.com). However, low production rates of lipopeptides by microbes lead to high costs of drugs and seriously restrict the application of lipopeptides in various settings. Therefore, it is necessary to improve the production of lipopeptides for their better applications.

Strategies for fermentation optimization and strain improvement are used to increase the bacterial lipopeptide production. To maximize the metabolite yield, the most suitable fermentation medium (nitrogen, carbon, trace element, etc.) and conditions (temperature, time, pH, etc.) must be identified and optimized [26]. Statistical approaches have been used to analyze the interactive effects of these parameters for optimization [22, 27, 28]. Plackett–Burman design (PBD) is a two-level factorial design that can quickly screen for significant factors [29]. The parameters screened by PBD can be further optimized by Box-Behnken design (BBD) [30] and response surface methodology (RSM) [31]. Combinations with these optimization methods have been used in a few studies to improve the lipopeptide production. For example, the yield of iturin A in *Bacillus amyloliquefaciens* LL3 was increased to 99.73 mg/L through above three methods [22]. Moreover, the natural strain screening and genetic engineering strategies are also effective ways to increase lipopeptide production [32, 33]. Transposon mutagenesis is a powerful genetic tool to screen regulators of secondary metabolites in bacteria [34, 35]. PhaR was identified as a negative transcriptional regulator by transposon library screening in *Streptomyces roseosporus*. The production of daptomycin in Δ*PhaR* was increased 6.14-fold, compared to that of the wild type strain [36].

In a previous study, we reported that the biocontrol agent *P. agglomerans* ZJU23, which was isolated from the *Fusarium* fruiting body microbiome, exhibited potent biocontrol efficacy against FHB and mycotoxin production. Further inestiagtion of its mode of action revealed that ZJU23 secreted the bioactive lipopeptide HA to inhibit fungal growth by directly disrupting ergosterol-containing lipid rafts [13]. However, few studies so far have focused on the optimization of HA production and regulation of HA biosynthesis [37]. In this study, we combined multiple optimization techniques to design a fermentation medium and process specifically for HA production. A Tn5 transposon library was constructed to screen for negative regulators of HA biosynthesis. Combining fermentation optimization and strain engineering, HA production was increased 5.39-fold, from 87.42 mg/L in the initial medium to ~ 470 mg/L.

## Results

### Screening of different media components and single-factor experiments

A total of six different media were assessed to identify the most suitable basis for further optimization of fermentation conditions  (Additional file 1: Table S1). After 72 h of incubation, HA was able to be detected in all tested media, but the yields were significantly different (Additional file 2: Fig. S1). The highest yield of HA (~ 87 mg/L) was achieved with King’s B (KB) medium [38], which was higher than in the TRIS-buffered chemically defined medium for HA production (TA, ~ 33 mg/L) [37]. Therefore, KB medium was selected as the base medium for further optimization.

**Fig. 1:**
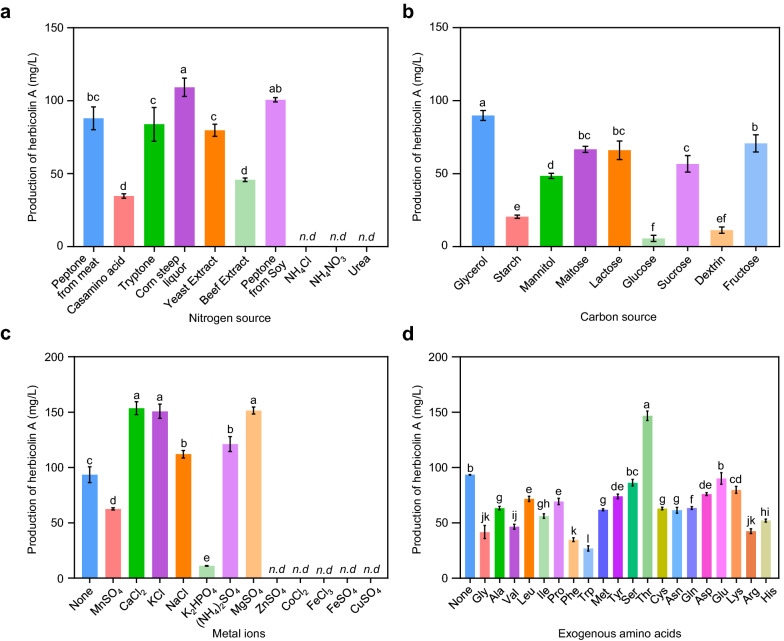
Effects of different medium compositions on HA production. The wild type strain ZJU23 was grown in a modified medium based on KB by using different nitrogen sources at a final concentration of 10 g/L (**a**), carbon sources at a final concentration of 15 g/L (**b**), metal ions at a final concentration of 1 mM (**c**), or amino acids at a final concentration of 10 mM (**d**). The concentration of HA in the individual medium was quantified by HPLC analysis after 72 h of incubation from *n* = 3 biologically independent samples (mean ± s.e.m.). Different letters indicate significantly different groups (*P* < 0.05, ANOVA and Fisher's LSD test). *Nd* not detectable

Subsequently, the effects of various nitrogen sources, carbon sources, metal ions, and amino acids on HA production were evaluated based on KB medium by a single-factor test. Nitrogen sources are critical for microbial growth and fermentation of desire compounds [39]. The effects of nine different nitrogen sources on HA production were examined by replacing peptone in KB medium while maintaining a constant level of nitrogen (10 g/L). The results indicated that corn steep liquor and peptone from soy were more effective for HA fermentation, with HA yields reaching 109.21 mg/L and 100.68 mg/L, respectively (Fig. 1a). HA was not detectable, when NH_4_Cl, NH_4_NO_3_, or urea was used as the sole nitrogen source. Thus, corn steep liquor was selected as the nitrogen source for further medium optimization. Next, the effects of 9 different carbon sources on HA production at a final concentration of 15 g/L were evaluated and it was found that medium supplemented with glycerol as carbon source led to the highest yield of HA production at 89.90 mg/L (Fig. 1b). We continued to screen metal ions (with a final concentration of 1 mM) that are considered as critical factors for the biosynthesis of secondary metabolites [40, 41]. In agreement with that, our results indicated that metal ions also play an important role in HA biosynthesis. CaCl_2_, KCl, and MgSO_4_ displayed strong positive effects on the production of HA, reaching 153.65 mg/L, 150.82 mg/L, and 151.58 mg/L, respectively. In contrast, supplementation of ZnSO_4_, CoCl_2_, FeCl_3_, FeSO_4_ or CuSO_4_ in the medium at the same concentration completely inhibited the HA biosynthesis (Fig. 1c). As a lipopeptide antibiotic, HA contains threonine, leucine, glutamic acid, glutamine, and arginine in its structure [13, 42].  The availability of specific amino acids might therefore be important for HA biosynthesis. Thus, 20 amino acids were selected as exogenous additives in the medium at a final concentration of 10 mM and their corresponding effects were tested. As shown in Fig. 1d, the production of HA reached the highest level (180 mg/L), when threonine (Thr) was added. Based on these observations, corn steep liquor, glycerol, CaCl_2_ and threonine were selected as the main medium components for further medium optimization.

### Identification of key fermentation factors via Plackett-Burman design

The Plackett-Burman design (PBD) was used to identify key factors that influence HA production. The impacts of each of the initially test factors with different concentration gradients and other four fermentation parameters (time, temperature, initial pH, and inoculation) were preliminary investigated (Additional file 3: Fig. S2). The results served as a basis for the PBD assessment (Additional file 1: Table S2). The PBD matrix constituted of 12 experimental runs and the corresponding HA are shown in Table 1. The obtained results that HA production varied from 89.40 to 183.36 mg/L; the highest HA yield was detected in Run 11.

**Table 1:** Plackett–Burman design (8 factors and 2 levels) for identifying main factors in HA production

Factor		A Corn steep liquor	B Glycerol	C CaCl_2_	D Threonine	E Time	F Temperature	G Initial pH	H Inoculation	Response HA (mg/L)
Std	Run									
9	1	− 1	− 1	− 1	1	1	1	− 1	1	169.70
7	2	− 1	1	1	1	− 1	1	1	− 1	89.40
1	3	1	− 1	1	− 1	− 1	− 1	1	1	106.89
11	4	− 1	1	− 1	− 1	− 1	1	1	1	88.59
3	5	− 1	1	1	− 1	1	− 1	− 1	− 1	106.01
8	6	− 1	− 1	1	1	1	− 1	1	1	96.79
4	7	1	− 1	1	1	− 1	1	− 1	− 1	158.49
12	8	− 1	− 1	− 1	− 1	− 1	− 1	− 1	− 1	108.30
5	9	1	1	− 1	1	1	− 1	1	− 1	108.50
2	10	1	1	− 1	1	− 1	− 1	− 1	1	141.67
6	11	1	1	1	− 1	1	1	− 1	1	183.36
10	12	1	− 1	− 1	− 1	1	1	1	− 1	118.97

**Fig. 2:**
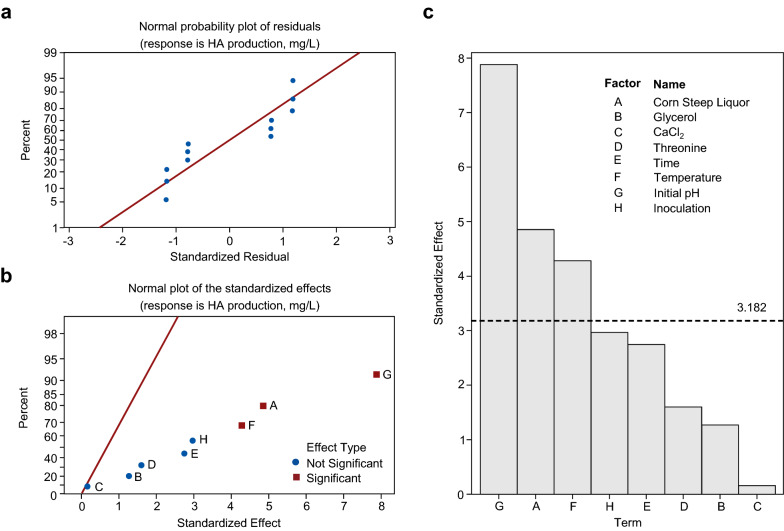
Response to the effects of standardized residuals and standardized effects on HA production. **a** Normal probability plot of the standardized residuals for HA production. **b** Half normal probability plot of the standardized effects for HA production. **c** Pareto chart ranking the standardized effects of the factors for HA production. Bars that are above the reference line (dotted line in figure) are statistically significant at a *P*-value < 0.05

The adequacy of the model and the significance of the tested parameters on HA production were evaluated by ANOVA (Table 2). The normal probability plot of the residuals indicated that the residuals are normally distributed and follow, approximately, a linear function (Fig. 2a). The first-order polynomial linear model of the PBD obtained by regression was as follows: HA production (mg/L) = 123.06 + 13.26A–3.47B + 0.43C + 4.37D + 7.50E + 11.70F–21.53G + 8.11H, where A, B, C, D, E, F, G, and H are designating corn steep liquor, glycerol, CaCl_2_, threonine, time, temperature, initial pH, and inoculation, respectively. The results indicated that all tested factors excluding glycerol and pH, had positive effects on HA production, which implies that HA production was improved by increasing the concentration of corn steep liquor, CaCl_2_, threonine (mM), or incubation time, temperature and inoculation. As shown in Table 2, the *P* value of the regression model was 0.023 (*P* < 0.05), indicating that the model was sufficient and significant. The coefficients of the determined R^2^ and adjusted R^2^ were 0.9765 and 0.9138, respectively, indicating the reliability of the model and therefore can be used to interpret the results of response variability.

**Table 2:** Analysis of variance and regression analysis of Plackett–Burman design on the HA production

Source	Degree of freedom	Adjusted sum of squares	Adjusted Mean square	*F*-value	*P*-value
Model	8	11,154.5	1394.32	15.57	0.023
A-Corn Steep Liquor	1	2109.7	2109.68	23.56	0.017
B-Glycerol	1	144.3	144.31	1.61	0.294
C-CaCl_2_	1	2.3	2.26	0.03	0.884
D-Threonine	1	229.2	229.21	2.56	0.208
E-Time	1	674.9	674.88	7.54	0.071
F-Temperature	1	1641.4	1641.42	18.33	0.023
G-Initial pH	1	5563.5	5563.47	62.14	0.004
H-Inoculation	1	789.3	789.3	8.82	0.059
Error	3	268.6	89.54		
Total	11	11,423.2			
R^2^	0.9765	Adjusted R^2^	0.9138

The variables with confidence level above 95% (*P* < 0.05) were considered as significant factors. In Table 2, corn steep liquor, temperature and initial pH were selected as the main affecting factors on HA production with *P* at 0.017, 0.023 and 0.004, respectively. Moreover, significant factors were further confirmed by normal plotting of standardized effects (Fig. 2b). Three significant variables had higher percentages and were lying together on the right-hand side of the standardized effect line. From the pareto chart, the effects of these variables on the responses can be intuitively comprehended and the statistical significance was provided. The *t*-values of corn steep liquor, temperature and initial pH were above the *t*-value limit line (3.182), which further confirmed that these three factors were the main affecting factors for HA production (Fig. 2c). Therefore, corn steep liquor together with temperature and initial pH was selected for further optimization using Box-Behnken design.

### Optimization of fermentation parameters by Box–Behnken design

The Box–Behnken design (BBD) matrix is presented in Table 3 and each factor was tested at three levels (−1, 0, and + 1). The selected parameters were optimized through 15 experiments, and the center point of each experiment was repeated three times. A maximum HA production of 194.32 mg/L was obtained in the frame of all 15 experiments, when the strain ZJU23 was grown in medium containing corn steep liquor at a final concentration of 11.5 g/L, an initial pH of 6.5, and incubation at 23 °C. The coefficients of linear, quadratic, and interaction terms were estimated and listed in Table 4. The quadratic polynomial model obtained by regression was:

**Table 3:** Box-Behnken design for HA production

Factor			A Corn Steep Liquor (g/L)	B Initial pH	C Temperature (°C)	Response HA (mg/L)
Std	Run	Pattern				
8	1	+ 0 +	15.0	6.5	26	133.67
9	2	0 − −	11.5	5.0	20	177.72
15	3	0 0 0	11.5	6.5	23	169.99
14	4	0 0 0	11.5	6.5	23	171.62
10	5	0 + −	11.5	8.0	20	99.52
5	6	− 0 −	8.0	6.5	20	101.26
1	7	− − 0	8.0	5.0	23	104.05
3	8	− + 0	8.0	8.0	23	72.81
11	9	0 − +	11.5	5.0	26	106.53
13	10	0 0 0	11.5	6.5	23	171.10
7	11	− 0 +	8.0	6.5	26	96.23
2	12	+ − 0	15.0	5.0	23	194.32
12	13	0 + +	11.5	8.0	26	108.52
6	14	+ 0 −	15.0	6.5	20	166.50
4	15	+ + 0	15.0	8.0	23	107.26

**Table 4:** Variance analysis results of Box-Behnken design for HA production

Source	Sum of squares	Degree of freedom	Mean Square	*F*-value	*P*-value
Model	20,727.32	9	2303.04	32.19	0.0007
A-Corn Steep Liquor	6464.19	1	6464.19	90.36	0.0002
B-Initial pH	4729.58	1	4729.58	66.11	0.0005
C-Temperature	1251.49	1	1251.49	17.49	0.0086
AB	778.76	1	778.76	10.89	0.0215
AC	193.17	1	193.17	2.70	0.1613
BC	1607.56	1	1607.56	22.47	0.0051
A^2^	2302.63	1	2302.63	32.19	0.0024
B^2^	2557.28	1	2557.28	35.75	0.0019
C^2^	1708.63	1	1708.63	23.88	0.0045
Residual	357.69	5	71.54		
Lack of Fit	356.31	3	118.77	172.27	0.0058
Pure Error	1.38	2	0.6894		
Cor Total	21,085.01	14	R^2^	0.9830	
Std. Dev	8.46		Adjusted R^2^	0.9525	
Mean	132.07		Predicted R^2^	0.7295	
C.V. %	6.40		Adeq Precision	15.2738	

HA production (mg/L) = 170.9 + 28.4A–24.3B–12.5C–14AB–6.9AC + 20BC—25A^2^–26.3B^2^–21.53C^2^, which was used to calculate the predicted production as a function of corn steep liquor (A), temperature (B), and initial pH (C).

Corn steep liquor, temperature, and initial pH were observed to be significant factors with *P* values of 0.0002, 0.0005, and 0.0086, respectively (Table 4). Statistical evaluation (*P* = 0.0007 < 0.01) indicated that the model is reliable. The regression coefficients R^2^ and Pred R^2^ were 0.9830 and 0.7295 respectively, which are reasonably consistent with Adj R^2^ (0.9525). The model terms A, B, C, AB, BC, A^2^, B^2^, and C^2^ were identified as significant terms.

**Fig. 3:**
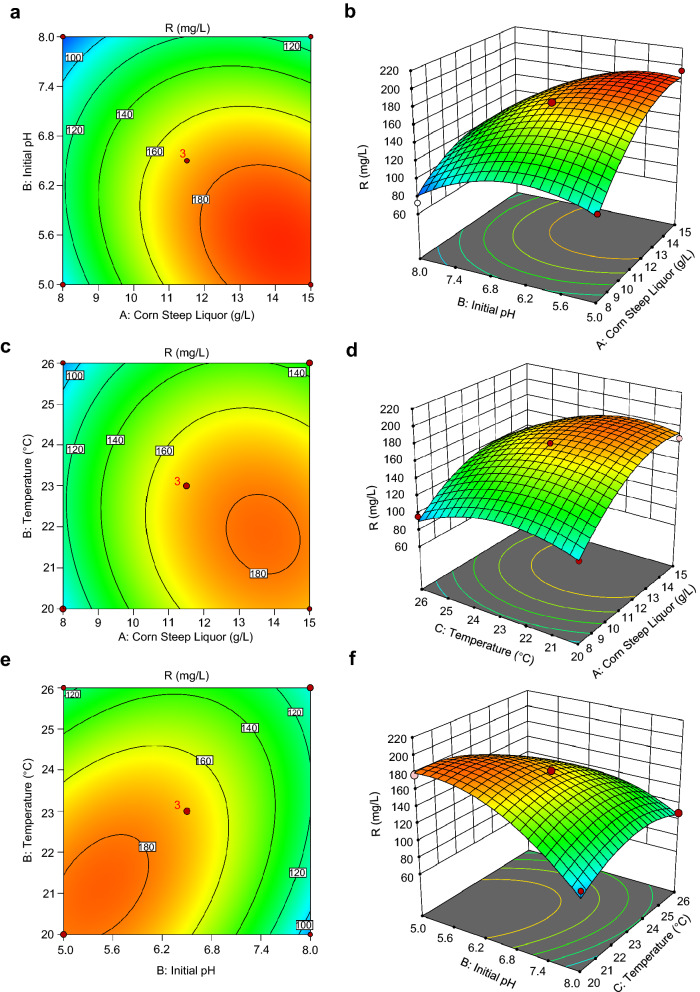
Two-dimensional contour plots and three-dimensional response surfaces for effect evaluation. Interaction of corn steep liquor and initial pH (**a**, **b**), corn steep liquor and temperature (**c**, **d**), and initial pH and temperature (**e**, **f**). **a**, **c**, **e** are the two-dimensional contour plots and **b**, **d**, **f** are the three-dimensional response surfaces

These three significant influence factors were further analyzed with the response surface methodology (RSM) to obtain the composition of the optimal fermentation medium by establishing a mathematical model. RSM results indicated that corn steep liquor (A), temperature (B), and initial pH (C) can adequately describe the response. The constructed 2D contour plots and the 3D response surface plots further confirmed that the production was mainly depended on different combinations of two parameters, i.e., corn steep liquor and initial pH (Fig. 3a, b), corn steep liquor and temperature (Fig. 3c, d), and initial pH and temperature (Fig. 3e, f), while holding the other factor at 0 level. The three factors optimized by RSM were as follows: corn steep liquor (A), 14.9 g/L; temperature (B), 20.3 °C; initial pH (C), 5.0. Among the three factors with significant effects, the initial pH was found to be the most pivotal factor affecting the production of HA, followed by corn steep liquor and temperature.

### Validation of the model and optimal conditions

According to the results obtained by BBD and RSM, the maximum HA yield was predicted to be 204.5 mg/L with calculated values for the factors: corn steep liquor, 14.9 g/L; temperature, 20.3 °C; initial pH, 5.0. To further evaluate and test the model, the strain ZJU23 was cultured in the optimized medium on a shaker (180 rpm) at 20.3 °C and the HA production was quantified after 60 h of incubation. The HA yield was 211.10 mg/L under the specified conditions, which was very close to the predicted yield. This result indicated that the response quadratic polynomial model can adequately reflect the expected optimization. HA production was significantly increased in the optimized medium when compared with the basic medium KB. Taken together, the determined compositions of the optimized medium for HA production with *P. agglomerans* ZJU23 was as follows: corn steep liquor, 14.9 g/L; glycerol, 10 g/L; CaCl_2_, 1 mM; threonine, 10 mM; initial pH 5.0. The defined medium was termed as HAI medium (HA induction medium). HA production reaches the maximum when the inoculated HAI medium (initial concentration of bacterial cells: 10^6^ CFU/mL) was cultured on a shaker (180 rpm) at 20.3 °C for 60 h.

### Inactivation of the transcriptional repressor LrhA enhanced HA production

**Fig. 4:**
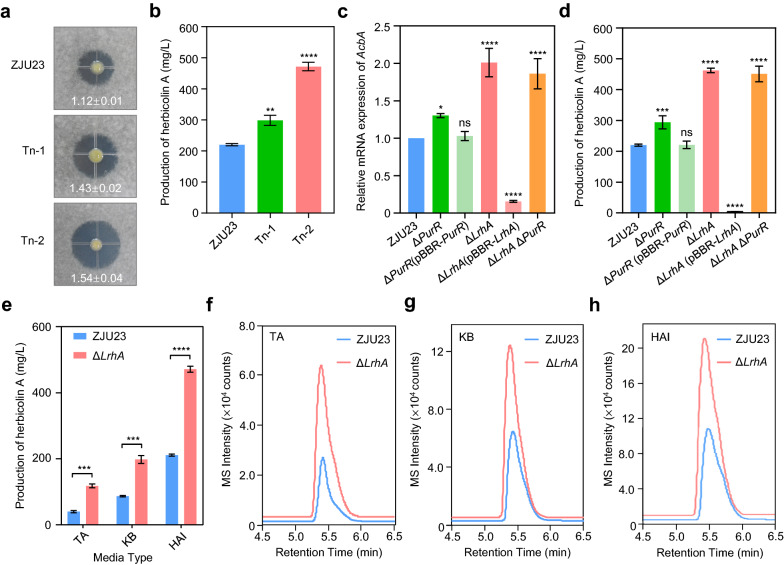
LrhA negatively regulates HA production in *P. agglomerans* ZJU23. **a** Inhibition activity of the wild type strain ZJU23, and transposon mutants Tn-1 and Tn-2 against the mycelial growth of *F. graminearum.* Diameters of inhibition zones were measured after 18 h of co-culture. Each experiment was repeated three times. **b** The HA production of ZJU23, Tn-1 and Tn-2. **c** Relative mRNA expression of the HA biosynthetic gene *AcbA* in ZJU23, mutants and complementation strains. Bacterial cells were harvested after 18 h of incubation and subjected to RNA extraction for quantification of the expression of *AcbA* by RT-qPCR. The 16S rRNA gene was used as an internal control. The expression in ZJU23 was set as 1.0. Data presented are the mean ± s.e.m. from three biological replicates. Differences between groups were analyzed using Student’s *t* test (paired, two-sided). *P < 0.05; ****P < 0.0001. **d** HA production of all tested strains. Strains were grown in HAI medium for 60 h. Data presented are the mean ± s.e.m. from three biological replicates. Differences between groups were analyzed using Student’s t test (paired, two-sided). **P < 0.01; ***P < 0.001; ****P < 0.0001. **e** Comparison of the HA production of ZJU23 and Δ*LrhA* fermented using TA, KB or HAI medium. Data presented are the mean ± s.e.m. from three biological replicates. Differences between groups were analyzed using Student’s t test (paired, two-sided). ***P < 0.001; ****P < 0.0001. **f**–**h** HPLC–MS quantification of the HA production in ZJU23 and Δ*LrhA* incubated in TA, KB, HAI medium, respectively

To further improve HA production by genetically modifying the wild type strain ZJU23, we constructed a random mutagenesis library for screening negative regulators of HA biosynthesis. More than 12,000 Tn5 transposon mutants were picked and inoculated on potato dextrose agar (PDA) medium supplemented with conidia of the fungal pathogen *F. graminearum*. After antifungal activity screening, two transposon mutants, termed as Tn-1 and Tn-2, displayed larger antifungal inhibition zones, when compared to the wild type strain (Fig. 4a). The HA production of these two transposon mutants was further quantified in HAI medium. Results indicated that the HA production was significantly enhanced in the mutants and reached 298.5 mg/L in Tn-1 and 472.0 mg/L in Tn-2, while the HA yield was 220.3 mg/L in the wild type (Fig. 4b). This implies that the transposon disrupted genes in Tn-1 and Tn-2 might be repressors for HA biosynthesis in ZJU23.

For a detailed assessment, these two transposon mutants were subjected to whole-genome re-sequencing for mapping the insertion sites in ZJU23. Results revealed that the transposon insertion sites were located within the open reading frames of ORF22 in Tn-1 and ORF2643 in Tn-2, respectively. Amino acid sequence analysis suggested that the ORF22 gene encodes a putative *LacI* family transcriptional repressor, which showed approximately 70% amino acid sequence identify to PurR of *Erwinia amylovora*, PtxS of *Klebsiella pneumoniae* and other hits (Additional file 4: Fig. S3a). The ORF2643 gene encodes a putative LysR-type DNA-binding transcriptional repressor, LrhA (Additional file 4: Fig. S3b). Its homologues were previously reported to repress biosynthesis of secondary metabolisms in *Photorhabdus* and *Xenorhabdu* [43–45]. The two identified genes were named *purR* and *lrhA* for further investigation.

To confirm the roles of PurR and LrhA on HA production, we constructed single deletion mutants, double deletion mutants, and corresponding complementation strains of the two genes. The mRNA expression levels of genes in the biosynthetic gene cluster AcbA-AcbJ [13] were determined by qRT-PCR in all tested strains using the *acbA* as a representative gene. The expression of *acbA* was significantly increased in both single and double deletion mutants (Fig. 4c). The reduced *acbA* expression was re-established in the complementation strain Δ*PurR* (pBBR-*PurR*). Notably, the Δ*LrhA* (pBBR-*LrhA*) displayed lower expression of *acbA* than that in the wild type strain, which might be caused by the plasmid pBBR1-MCS5 using for complementation construction. It is a high-copy-number plasmid resulting in the overexpression of LrhA. In agreement with mRNA expression of HA biosynthetic genes, the HA production was substantially enhanced in all mutants, but significantly reduced in the Δ*LrhA* (pBBR-*LrhA*), in comparison with that in the wild type strain ZJU23 (Fig. 4d). Notably, the double mutant Δ*LrhA*Δ*PurR* presented similar effects on the biosynthetic gene expression and HA production when compared to the single mutant Δ*LrhA* (Fig. 4c, d). This implied that LrhA is a master repressor for HA biosynthesis.

In addition, HA production in Δ*LrhA* was also enhanced in TA (~ 2.9 fold) [37] and the base medium KB (~ 2.3 fold) (Fig. 4e–h). Overall, a HA yield of 471 mg/L was reached by combining the optimized medium HAI with the repressor mutant Δ*LrhA; *this was significantly higher than that of the wild type strain fermented in HAI (~ 210 mg/L).

### Optimization of HA production increased the biocontrol efficacy against fungal diseases in crops

**Fig. 5:**
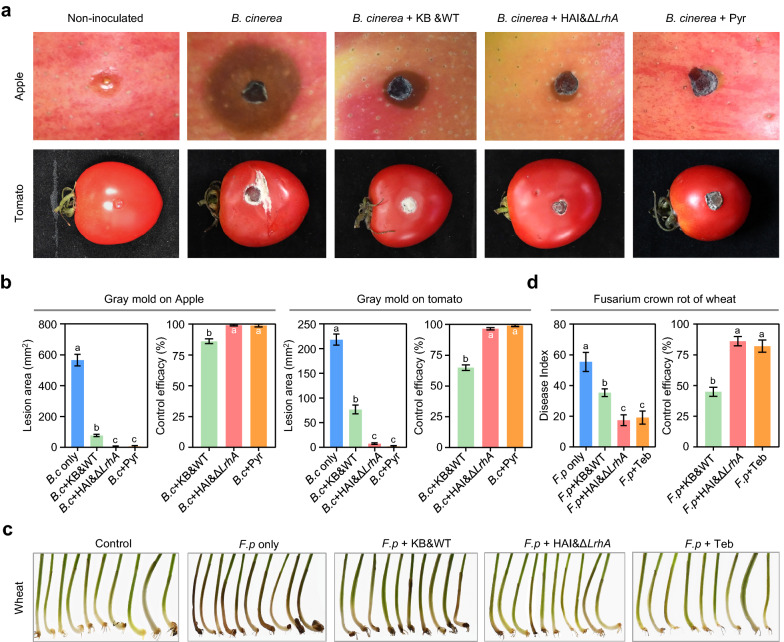
Biocontrol efficacy of *P. agglomerans* ZJU23 was enhanced after fermentation optimization and strain engineering. **a** Control effect on gray mold of apple and tomato caused by *B. cinerea* (*B.c*) using the bacterial suspension of the wild type strain ZJU23 fermented in KB (treatment KB&WT, before optimization) or Δ*LrhA* fermented in HAI (treatment HAI&Δ*LrhA*, after optimization). Apple and tomato inoculated with *B. cinerea* only was used as the non-treatment control, and pyrimethanil (Pyr) was used as a chemical fungicide control. The representative images in each treatment were obtained after 3 days of treatment. **b** Lesion area and disease control efficacies of different treatments against gray mold disease. **c** Control effect on Fusarium crown rot of wheat caused by *F. pseudograminearum* (*F.p*). Wheat seedlings treated by *F. pseudograminearum* only were used as the non-treatment control, and tebuconazole (Teb) was used as a chemical fungicide control. The representative images in each treatment were shown after 14 days of treatment. (d) Disease index and control efficacies of different treatments against Fusarium crown rot of wheat. Data presented are the mean ± s.e.m. from three biological replicates. Different letters indicate significantly different groups (*P* < 0.05, ANOVA and Fisher's LSD test)

HA is the main antifungal compound produced by ZJU23 [13]. To determine whether the increased HA production that was achieved by combing fermentation optimization and genetics manipulations can enhance the biocontrol efficacy against crop fungal diseases, biocontrol experiments in a growth chamber were conducted. The efficacy was assessed with test systems that included gray mold of apple and tomato, and Fusarium crown rot of wheat, caused by *Botrytis cinerea* and *Fusarium pseudograminearum*, respectively. Treatments with the bacterial suspension of the wild type strain ZJU23 fermented in KB (treatment KB&WT, before optimization) as well as Δ*LrhA* fermented in HAI (treatment HAI&Δ*LrhA* after optimization) significantly suppressed disease progression of *B. cinerea* strain B05.10 on apple and tomato, and of *F. pseudograminearum* strain F303 on wheat seedlings (Fig. 5a, c). Importantly, the biocontrol efficacy in the treatment HAI&Δ*LrhA *was significantly higher than that in the treatment KB&WT against the tested fungal diseases, almost reaching the effect of chemical fungicide treatments (Fig. 5b, d).

**Fig. 6:**
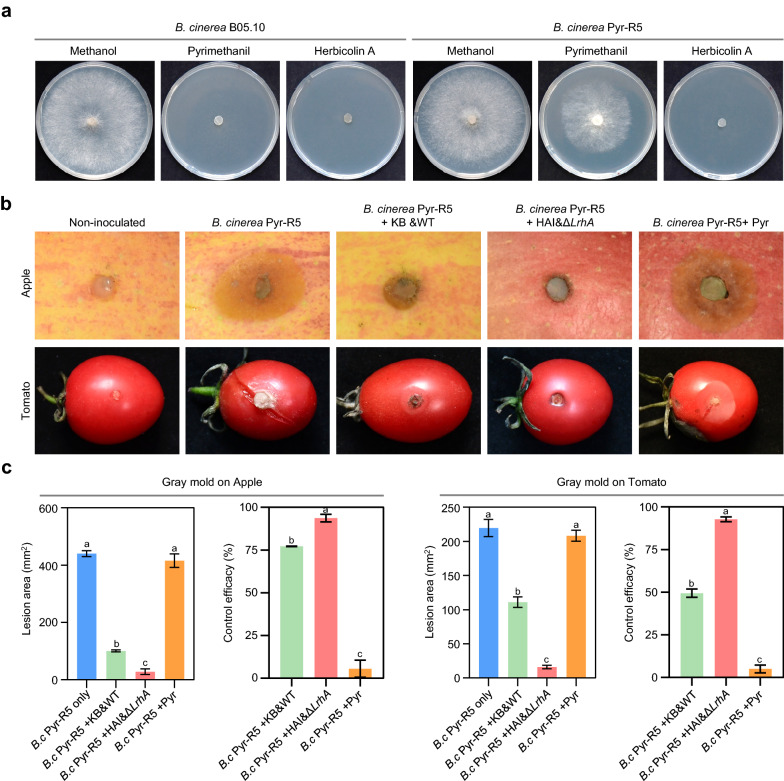
Optimization of HA production increased the biocontrol efficacy against gray mold caused by a pyrimethanil resistant strain. **a** Mycelial growth inhibition of pyrimethanil and HA towards *B. cinerea* (*B.c*) pyrimethanil-sensitive strain B05.10 and pyrimethanil-resistant strain Pyr-R5. The final concentration of pyrimethanil or HA was 5 μg/ml. Images were taken after 5 d of incubation. The solvent methanol was used as a control. The experiment was repeated three times with similar results. **b** Control effect on gray mold of apple and tomato caused by *B.c* Pyr-R5 using the bacterial suspension of the wild type strain ZJU23 fermented in KB (treatment KB&WT, before optimization), Δ*LrhA* fermented in HAI (treatment HAI&Δ*LrhA*, after optimization) or pyrimethanil (Pyr). Apple and tomato inoculated with *B.c* only was used as the non-treatment control. The representative images in each treatment were obtained after 3 d of treatment. **c** Lesion area and disease control efficacies of different treatments against gray mold disease caused by *B.c* Pyr-R5. Data presented are the mean ± s.e.m. from three biological replicates. Different letters indicate significantly different groups (*P* < 0.05, ANOVA and Fisher’s LSD test)

Biocontrol agents have been proposed to be an alternative approach for managing fungicide resistance in plant pathogens. Therefore, we also assessed whether optimization of HA production can increase the biocontrol efficacy against fungicide resistant pathogens. The pyrimethanil resistant *B. cinerea* strain Pyr-R5 was for this purpose. We first investigated whether HA was able to inhibit the mycelial growth of Pyr-R5. As shown in Fig. 6a, Pyr-R5 was susceptible to HA at the same level as the non-resistant strain *B. cinerea* B05.10, but showed high resistance towards pyrimethanil. Next, we conducted the biocontrol experiments in a growth chamber against gray mold of apple and tomato caused by Pyr-R5. Both KB&WT and HAI&*ΔLrhA* treatment were highly effective against the disease caused by Pyr-R5. Moreover, increased HA production of the HAI&*ΔLrhA* treatment substantially enhanced the biocontrol efficacy in comparison to the KB&WT treatment. In contrast, pyrimethanil was unable to control the grey mold disease caused by Pyr-R5 on tomato and apple (Fig. 6b, c). Taken together, these results suggested that a combination of fermentation optimization and strain genetic modification can substantially improve the biocontrol efficacy of *P. agglomerans* ZJU23 against crop fungal diseases, and provides a viable alternative for managing fungicide resistance.

## Discussion

The lipopeptide HA producted by *P. agglomerans* (former name *Erwinia herbicola*) strains is a potential antifungal drug under agricultural and clinical settings [13, 46]. Optimization of medium compositions and fermentation conditions can be an effective method to improve the production of antifungal lipopeptides [20, 26]. The only publication on HA fermentation was reported in 1991. The HA  ~ 150 mg/L at low cultivation temperatures in TA medium as presented by Greiner and Winkelmann [37]. They found that high concentrations of Tris-buffer, low temperature (16 °C), glutamic acid (12 mM), and fermentation time (~ 60 h) were important factors for HA production in *E. herbicola* strain A111 [37]. In this study, we combined multiple optimization techniques, including single-factor experiments, PBD, BBD and RSM, to improve HA production reaching yields higher than 200 mg/L. Our results indicated that corn steep liquor, glycerol, CaCl_2_, and threonine are positively influencing HA production in the single-factor experiments. Among these medium constituents, corn steep liquor as the sole nitrogen source is extensively used in microbial fermentation, but its detailed imolications on the biosynthesis of desired products were not identified yet despite extensive efforts [39, 47, 48]. Lipopeptides are formed by a fatty acid (between C12 and C18) linked to a peptide chain (from 4 to 12 amino acids) [49]. Previous studies reported that supplementation of amino acids present in the peptide chain to the fermentation medium can significantly improve the yield of desired lipopeptides [50, 51]. In agreement with that, we here found that threonine was able to significantly enhance HA production in ZJU23 among the 20 tested amino acids. This is not consistent with the finding that glutamic acid is critical for HA biosynthesis in *E. herbicola* strain A111 [37]. These variations might be due to different strain backgrounds and nitrogen sources used in the conducted experiments. Our results suggested that nitrogen source, temperature, and initial pH are significant factors affecting HA production, and led to the design of an optimized medium and conditions for HA fermentation.

In our previous study, the biosynthetic gene cluster AcbA-AcbJ for HA production in ZJU23 was described for the first time [13]. However, the regulation of HA biosynthesis was unknown. A variety of genetic approaches were used to enhance the production of lipopeptides, including genetic engineering or random mutagenesis for repressor screening [33, 52, 53]. Here, we constructed a transposon library to screen negative regulators of HA biosynthesis, and obtained two repressors for HA production, PurR and LrhA. LrhA homologues were identified as a LysR-type transcriptional repressors that regulate secondary metabolites in various bacterial genera. For example, ScmR in *Burkholderia thailandensis* E264 was shown to limit the production of burkholdac A and malleilactone [54]. In *Pseudomonas aeruginosa*, the LysR-type protein MvfR was reported to regulate quinolone signal production [55]. In addition, in *Erwinia carotovoras*, LrhA was identified as a negative regulator of the quorum-sensing signal, *N*-(3-oxohexanoyl)-L-homoserine lactone [56]. Disruption of the *LrhA* gene in ZJU23 significantly increased the expression of biosynthetic genes and subsequently resulted in an enhancement of HA production. Therefore, we proposed that LrhA is a master repressor to regulate the expression of AcbA-AcbJ in *P. agglomerans* ZJU23.

Various antimicrobial peptides have emerged as potential candidates for developing new antifungal therapeutics over the past years and are often characterized by negligible host toxicity and low resistance formation rates [21]. Lipopeptides constitue a specific class of microbial secondary metabolites and harbor diverse biological functions, especially antimicrobial and anticancer activities [19, 20, 57–62]. Lipopeptides can be employed for pharmaceutical, food-related, agricultural, and environmental protection applications [63, 64]. In the present study, we demonstrated that increased HA yields in the ZJU23 deletion mutant result in control effects of fungal pathogens that are comparable to synthetic fungicides. This highlights its applicability for crop protection. HA belongs to the cyclic lipopeptides and its structure consists of a peptide ring of eight amino acids where a fatty acid chain, a dehydrobutyric acid, and a sugar moiety are attached [13, 46]. HA is known to have a broad antifungal spectrum and a high control efficiency towards phytopathogenic fungi. However, similar to other lipopeptides, lower production rates and high costs are the main limiting factors for its commercialization. Therefore, HA production will likely require further optimization to facilitate its commercialization in the future.

## Conclusions

In this study, production of HA in the biocontrol agent *P. agglomerans* ZJU23 with high yield was achieved by combining fermentation optimization and strain engineering. The defined HAI medium was obtained for HA fermentation, and the repressor for HA production, LrhA, was identified. HA production reached ~ 471 mg/mL with the Δ*LrhA* mutant under optimized fermentation conditions, which is about 5.4 times higher than the initial concentration, and significantly enhanced its biocontrol efficacy against the tested fungal diseases. These findings provide an extended basis for large-scale production of HA and promote the biofungicide development based on ZJU23 and HA in future.

## Methods

### Strains, plasmids, and culture conditions

*P. agglomerans* ZJU23 was isolated from a *Fusarium* fruiting body and deposited in a strain collection [13]. *Escherichia coli* DH5α was used for plasmid construction and transformation. All bacterial strains and plasmids used in this study are listed in Additional file 1: Table S3. ZJU23 and its derivatives were cultivated in Luria–Bertani (LB) [65] broth at 30 °C, while *E. coli* was cultivated at 37 °C. Antibiotics were used when required. The non-resistant strain *B. cinerea* B05.10, *F. pseudograminearum* F303, and pyrimethanil resistant *B. cinerea*Pyr-R5 were grown on PDA and incubated at 25 °C.

### Selection of the base medium and single-factor experiments

For HA production, an overnight culture was subsequently adjusted to an optical density at 600 nm (OD_600_) of 1.0 and used to inoculate 100 mL fermentation medium in 250 mL flasks at a ratio of 1:1000 (100 μL; the initial inoculation concentration was 1 × 10^6^ CFU/mL). Then flasks were cultured at 25 °C with shaking at 180 rpm for 72 h. A total of six media were screened for HA production, including TA, LB, KB, TSB (Tryptic Soy Broth) [66], NB (Nutrient Broth) [67] and WA (Warkingsman’s medium) [15]. The compositions of the individual media are shown in Additional file 1: Table S1. KB medium was selected as the base medium for further investigation. Subsequently, the effects of different nitrogen sources, carbon sources, inorganic salts, and amino acids on HA production were tested. These components were used individually to replace corresponding components in the determined base medium with the same concentration, whereas other ingredients remained unchanged. To further improve the production of HA, eight fermentation parameters, including A: corn steep liquor, B: glycerol, C: CaCl_2_, D: threonine, E: time, F: temperature, G: initial pH and H: inoculation, were evaluated. The following are the initial parameters: A, 15 g/L; B, 10 g/L; C, 1 mM; D, 10 mM; E, 72 h; F, 25 °C; G, 7.0; H, 1 × 10^8^ CFU.

### Plackett–Burman design

Plackett–Burman design was employed to identify significant fermentation parameters that are important for HA production. Twelve runs with eight variables (A–H) at two levels (−1 and + 1) were investigated based on the single-factor tests. The factors and the corresponding high and low levels are shown in Additional file 1: Table S2. The data were fitted with Minitab 17 Statistical Software at a significance level of *P* < 0.05.

### Box–Behnken design

Box-Behnken design was used to further optimize the three main affecting factors for HA production: corn steep liquor (A), initial pH (B), and temperature (C). Three factors with three levels (− 1, 0, 1) were investigated. The experimental design includes 12 factorial points and 3 center points. Table 3 lists the variable levels and response values. Concentrations of other medium components were as follows: glycerol, 10 g/L; CaCl_2_, 1 mM; threonine, 15 mM; time, 60 h; inoculation, 1 × 10^8^ CFU. The data were fitted by Design-Expert 12.0 (Stat-Ease Inc.) at a significance level of* P* < 0.05.

### RSM analysis and model validation

The response surface plot of the quadratic polynomial model was generated by varying one of the independent variables within the range of the experiment while keeping the other variables unchanged at the center point to understand the effect of the independent variable on the dependent variable. RSM ridge analysis was carried out using the statistical software Design-expert 12.0 (Stat-Ease Inc.) to predict the maximal HA yield. After the optimal parameters were predicted, validation experiments were conducted by fermentation in medium with an optimized composition.

### Quantification of HA via HPLC analysis

Quantitative analysis of HA was performed as previously described [13]. Briefly, 1 mL of the fermentation supernatant was collected and freeze-dried. The precipitate formed was resuspended with 1 mL methanol for HA extraction and the crude samples were subjected to HPLC analysis (Agilent Technologies 1100 Infinity) under the following conditions: C18 reversed-phase column [Agilent ZORBAX RX-C18 column (250 × 4.6 mm)] eluted with methanol/H_2_O (A/B) (1 mL/min, 30–90% A in 30 min, followed by 90–100% A in 10 min). The peak areas were used to quantify the production of HA according to the standard sample. To quantify HA more precisely, the extracted samples were analyzed by liquid chromatography-mass spectrometry (LC–MS).

### Construction of transposon mutants and gene deletion mutants

A transposon mutagenesis library and gene deletion mutants of ZJU23 were constructed as described previously [13]. Briefly, transposon mutants were generated by conjugation of recipient ZJU23 resistant to rifampicin and donor *E. coli* SM10_λ-pir_. Each transposon was screened in an inhibition zone assay against *F. graminearum.* The selected transposons were re-sequenced by Beijing Novogene Bioinformatics Technology Co., Ltd. to localize the insertion by comparing it with the wild type strain ZJU23. Gene deletion mutants were generated by using the λ-red recombinase method. The transformants were confirmed by PCR. Double mutant strains were generated using single mutants as a background strain as indicated. Complementation constructructions were generated as described previously [68]. The primers designed in this study for mutant strains and complementation constructions are listed in the Additional file 1: Table S4. The primer pair M13-F and M13-R were used for identification of pBBR-*LrhA* and pBBR-*PurR* plasmid construction [69].

### RNA preparation and quantitative reverse transcription PCR (qRT-PCR)

For real-time quantitative PCR (RT-qPCR), all strains were cultured with HI medium at 25 °C and 180 rpm for 18 h. Total RNA purification was performed by using RNAprep Pure Cell/ Bacteria Kit (TIANGEN, DP430) and reverse transcription was done using HiScript II Q RT SuperMix for qPCR (+ gDNA wiper) (Vazyme, R223-01) according to the manufacturer's instructions. RT-qPCR was performed via qRT-PCR using ChamQ SYBR qPCR Master Mix (Vazyme, Q311-02). The experiments were performed in independent biological triplicates and 16S rRNA of ZJU23 was used as the internal control. The mRNA fold change was estimated by the threshold cycle (*C*_*t*_) values of 2^−(ΔΔCt)^. The primers used for qRT-PCR assays are listed in Additional file 1: Table S4.

### Evaluation of biocontrol efficacy of the fermentation suspension

Biocontrol experiments with a fermentation suspension produced by ZJU23 in KB and Δ*LrhA* in HAI against gray mold caused by *B. cinerea and* Fusarium crown rot caused by *F. pseudograminearum* were conducted. The gray mold assay was performed as previously described [70]. The corresponding fermentation broth, pyrimethanil (50 mg/L) or water were sprayed on the tomato or apple surface. After 1 h air-drying, fresh mycelial plugs (6 mm in diameter) were inoculated. Pyrimethanil (50 mg/L) and clean water were used as fungicide treatment and negative control. Disease lesions were observed and measured 72 h after inoculation with the pathogenic fungus. The Fusarium crown rot assay was performed as described previously but with specific modifications [71]. *F. pseudograminearum* F303 conidia (1 × 10^6^ CFU/mL) were mixed with soil at 1:10 (W/W) for three days. Then wheat seeds (cultivar: Jimai 22) were planted into conidia-inoculated soil. Non-inoculated soil was used as a control. At the seventh day, 50 mL of the fermentation suspension were sprinkled on the wheat root. Tebuconazole (150 mg/L) and clean water were used as fungicide treatment and negative control. After 21 days, the roots were washed and the browning areas of the wheat stem base were observed. The disease index (DI) was calculated as previously described [72] but with modifications. Five evaluation classes ranging from 0 to 4, were applied for the disease index. The disease index corresponds to the percentage of the browning length of the first stem node (0 = 0, 1 = 1–25%, 2 = 26–50%, 3 = 51–75%, and 4 ≥ 75%). The disease index was calculated as follows in Eq. (2):

1 $${\text{DiseaseIndex}}\left( {{\text{DI}}} \right)\, = \,\left( {\sum \, \left( {{\text{number of diseased plants in each class}}\, \times \,{\text{each evaluation class}}} \right)} \right)/\left( {{\text{total number of investigated plants}}\, \times \,{\text{the highest disease index}}} \right)\, \times \,{1}00$$

The control efficacy was calculated as follows in Eq. (3):

2 $${\text{Control efficacy}}\, = \,\left( {{\text{disease index of control}} - {\text{disease index of treatment}}} \right)/ \, \left( {\text{disease index of control}} \right)\, \times \,{1}00\%$$

### Statistical analysis

All experiments in this study were repeated three times. Data presented are the mean ± standard errors. Differences between two groups were analyzed by Student’s t-test. Multiple comparisons were analyzed by one-way analysis of variance (ANOVA) followed by the least significant difference (LSD) multiple-range test.

## Supplementary Information

**Additional file 1: Table S1.** The component of different media used for basic medium screening. **Table S2.** List of levels and factors in the Plackett–Burman design experiments. **Table S3.** Strains and plasmids used in this study. **Table S4.** PCR primers used in this study.

**Additional file 2: Fig. S1.** Selection of a base medium for HA production.

**Additional file 3: Fig. S2.** Effects of different components in the medium and various fermentation parameters on the HA production.

**Additional file 4: Fig. S3.** Phylogenetic analysis based on the amino acid sequences of proteins encoded by ORF22 **a** and ORF2643 **b** in *P. agglomerans* ZJU23.

## Data Availability

All data generated or analyzed during this study are included in this published article [and the additional files].

## Abbreviations

PBD: Plackett–Burman design
BBD: Box-Behnken design
RSM: Response surface methodology
HA: Herbicolin A
