# Supplementary material for: Enhancement of herbicolin A production by integrated fermentation optimization and strain engineering in Pantoea agglomerans ZJU23
Source: Microb Cell Fact. 2023 Mar 13;22:50. doi: 10.1186/s12934-023-02051-z (PMC10012537; doi:10.1186/s12934-023-02051-z)
Supplement: Supplementary file 1 — Additional file 1: Table S1. The component of different media used for basic medium screening. Table S2. List of levels and factors in the Plackett–Burman design experiments. Table S3. Strains and plasmids used in this study. Table S4. PCR primers used in this study. [file 12934_2023_2051_MOESM1_ESM.docx]

**Table S1. The component of different media used for basic medium screening**

| **Medium** | **Components and contents** |
| --- | --- |
| **TA (**TRIS-buffered chemically defined medium for herbicolin A production**)** | K_2_HPO_4_, 8 g/L; (NH_4_)_2_SO_4_, 2 g/L; tri-Na-citrate, 0.5 g/L; MgSO_4_·7 H_2_O, 0.1 g/L; glucose, 20 g/L (autoclaved separately); TRIS-buffer, 24.2 g/L. |
| **LB** (Luria-Bertani Broth) | Tryptone, 10 g/L; yeast extract, 5 g/L; NaCl, 10 g/L. |
| **KB** (King‘s B Medium) | Peptone from meat, 10 g/L; glycerol, 15 g/L; K_2_HPO_4_, 1.5 g/L; MgSO_4_, 6 g/L. |
| **TSB** (Tryptic Soy Broth) | Tryptone 15 g/L; Soybean peptone 5 g/L; NaCl 5 g/L. |
| **NB (**Nutrient Broth**)** | Peptone from meat10 g/L; beef extract 3 g/L;  NaCl, 5 g/L. |
| **WA (**Warkingsman’s medium**)** | Peptone from meat 5 g/L; beef extract 3 g/L; NaCl, 5 g/L, glucose, 10 g/L. |

**Table S2.** **List of levels and factors in the Plackett-Burman design experiments**

| **Factor Symbol** | **Factor** | **Level** | |
| --- | --- | --- | --- |
|  |  | -1 | 1 |
| A | Corn Steep Liquor（g/L） | 8 | 12 |
| B | Glycerol（g/L） | 5 | 10 |
| C | CaCl_2_ (mM) | 0.3 | 1 |
| D | Threonine（mM） | 8 | 15 |
| E | Time（h） | 48 | 72 |
| F | Temperature (°C) | 20 | 25 |
| G | Initial pH | 5.0 | 8.0 |
| H | Inoculation (CFU/mL) | 1×10^7^ | 1×10^8^ |

**Table S3. Strains and plasmids used in this study**

| **Strains** | **Description** |
| --- | --- |
| *E. coli* |  |
| DH5α | Host strain for molecular cloning |
| SM10_λ-pir_ | thi thr leu tonA lacY supE recA::RP4=-2-Tc^R^ :  Mu l pir lysogen, oriT of RP4, Km^R^; Conjugal donor |
| *P. agglomerans* |  |
| ZJU23 | Wild-type strain *Pantoea agglomerans* ZJU23 |
| Δ*LrhA* | In-frame deletion of *orf2643*; Km^R^ |
| Δ*LrhA* (pBBR-LrhA) | ΔLrhA harboring plasmid pBBR-LrhA; Km^R^; Gm^R^ |
| Δ*PurR* | In-frame deletion of *orf22*; Km^R^ |
| Δ*PurR*(pBBR-PurR) | ΔPurR harboring plasmid pBBR1-PurR; Km^R^; Gm^R^ |
| Plasmids |  |
| pKD46 | Temperature sensitive λ-red expressing plasmid |
| pKD4 | Template plasmid with Kan^R^ gene and FLP recognition target |
| pCP20 | FLP recombinase helper plasmid, ts-rep,Amp^R^, Cm^R^ |
| pSC123 | Suicide plasmid; Tn5 transposon; Km^R^ |
| pBBR1-MSC5 | Broad-host-range vector with Plac promoter |
| pBBR-LrhA | pBBR1-MCS5 cloned with *lrhA* fragment |
| pBBR-PurR | pBBR1-MCS5 cloned with *purR* fragment |

**Table S4. PCR primers used in this study**

| **Primer** | **Sequence (5’-3’)** | **Characteristics** |
| --- | --- | --- |
| *lrhA*-F | GCGTTTATTAAGGCAATCACGCGGGTATTGAGTACAACACGTTAGCGAAATgtctcaaaatctctgatgttac | PCR primers for amplification of Km fragment with 50 bp homologous arms of *lrhA* |
| *lrhA* -R | GATAACACCAGGGTAGTAGTTCGTAAAAATCTTATAAGTGAAGAAAAAACTTAGAAAAACTCATCGAGCATC |  |
| *lrhA* -ID-F | AATCGCTTCAGTCTGTAGGGT | PCR primers for the identification of  *lrhA* disruption mutants |
| *lrhA* -ID-R | GATAAAATGCTTGATGGTCGG |  |
| *lrhA*-N-F | GAACCGTCGGGATTATTGCTC | PCR primers for amplification of  *lrhA* fragment |
| *lrhA*-N-R | ATGGCGGAAATCGCCCAGGCACTCAAG |  |
| *purR*-F | ATGATCGTGCTCACGAAGCGAGCACGTAAACGGAAGGTTAAATCTGAAATtgtctcaaaatctctgatgttac | PCR primers for amplification of Km fragment with 50 bp homologous arms of *purR* |
| *purR*-R | GCTTTGCTTGTGCTAGTCTCTGCCTGGTTTCTTATTACGCAGGATATCTCTTAGAAAAACTCATCGAGCATC |  |
| *purR*-ID-F | AACTGGCGTGCTTCTTCAAC | PCR primers for the identification of *purR* disruption mutants |
| *purR*-ID-R | CAACCAAACCGTTATTCATTC |  |
| *purR*-N-F | GGCTGGAGCCGCGAACGATAAG | PCR primers for amplification of  *purR* fragment |
| *purR*-N-R | GTCGGGATGCGTGAAGAGGTGC |  |
| *LrhA*-Com-F | CGGACTAGTTTTATTGCAGGATTGTGAGCCGTAC | PCR primers for amplification of *lrhA* full-length fragment along with their upstream promoter regions |
| *LrhA*-Com-R | CGCCTCGAGGAGGCTTATTTATAAGCGTTTATTAAGGC |  |
| *PurR-C*om-F | CCGAA TTCGCGGTGGGCTGGTCG | PCR primers for amplification of *purR* full-length fragment along with their upstream promoter regions |
| *PurR*-Com-R | CGAAGCTTCCGGTTCCAAAGCAAAGC |  |
| M13-F | GCTTATTATCACTTATTCAGGCGTAG | PCR primers for identification of pBBR-*LrhA* and pBBR-*PurR* plasmid construction |
| M13-R | TTAGCTCACTCATTAGGCACCC |  |
| RT-*AcbA*-F | CGAATGGCAGGTAGAGCAGT | Quantitative real-time PCR primers for analysis of *AcbA* expression |
| RT-*AcbA*-R | GTCCAAGGTGAGTTCCGATG |  |
| RT-16S-F | GCTCGTGTTGTGAAATGTTGG | Quantitative real-time PCR primers for analysis of *16S* expression |
| RT-16S-R | CACTTTGTGAGGTCCGCTTG |  |
