# Supplementary figures and images for: Enhancement of herbicolin A production by integrated fermentation optimization and strain engineering in Pantoea agglomerans ZJU23
Source: Microb Cell Fact. 2023 Mar 13;22:50. doi: 10.1186/s12934-023-02051-z (PMC10012537; doi:10.1186/s12934-023-02051-z)

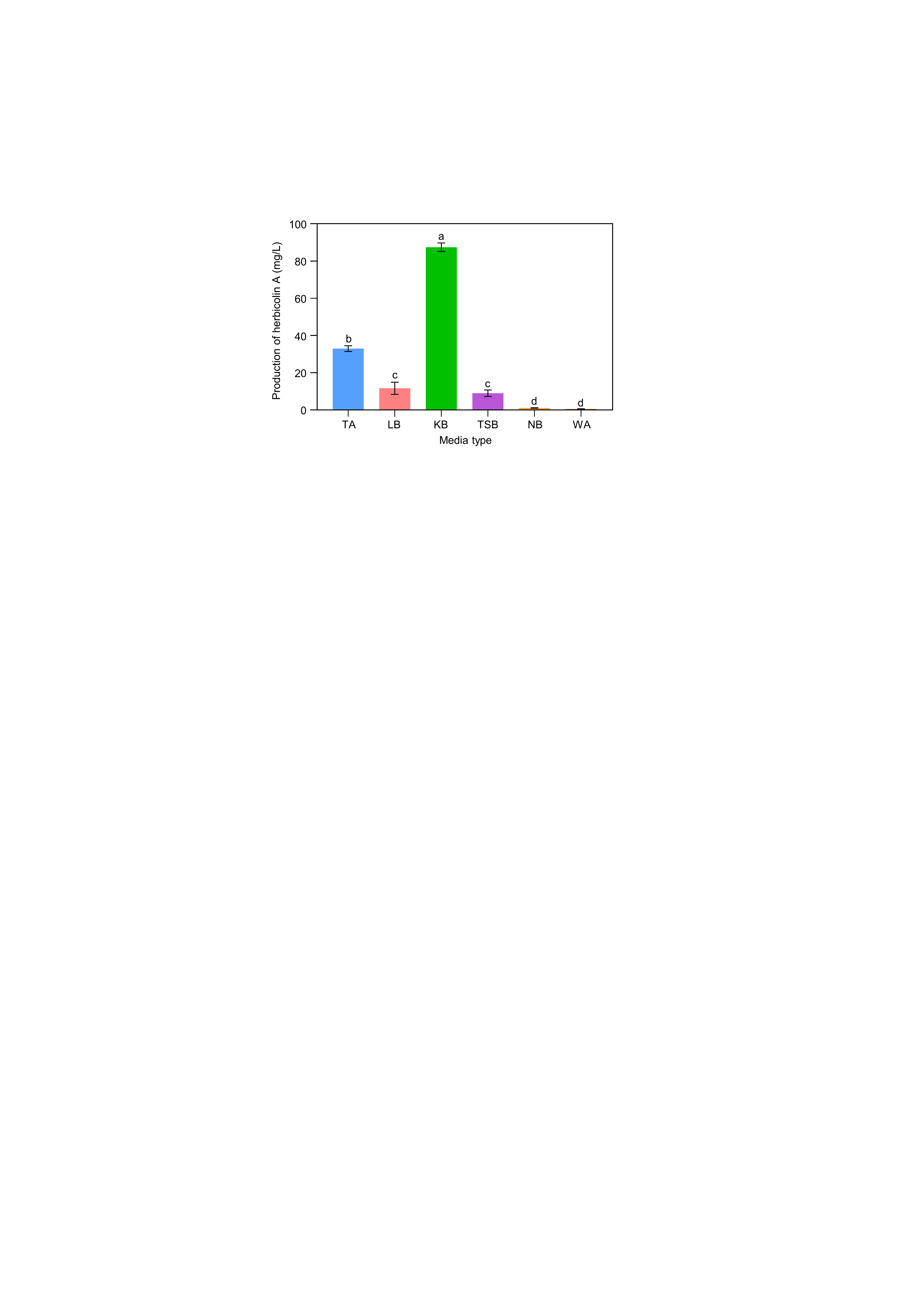

Supplement: Supplementary file 2 — Additional file 2: Fig. S1. Selection of a base medium for HA production. [file 12934_2023_2051_MOESM2_ESM.tif]

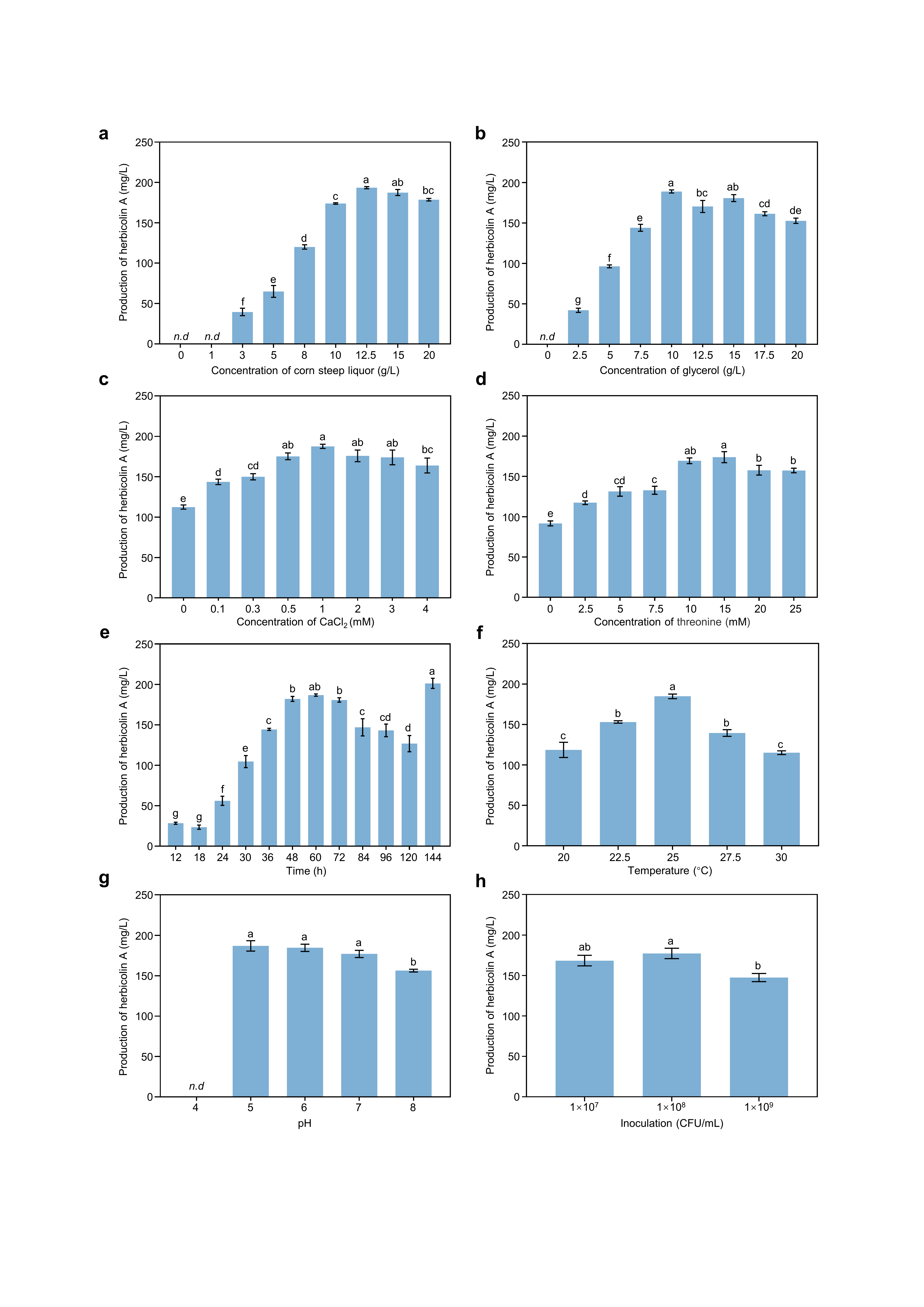

Supplement: Supplementary file 3 — Additional file 3: Fig. S2. Effects of different components in the medium and various fermentation parameters on the HA production. [file 12934_2023_2051_MOESM3_ESM.tif]

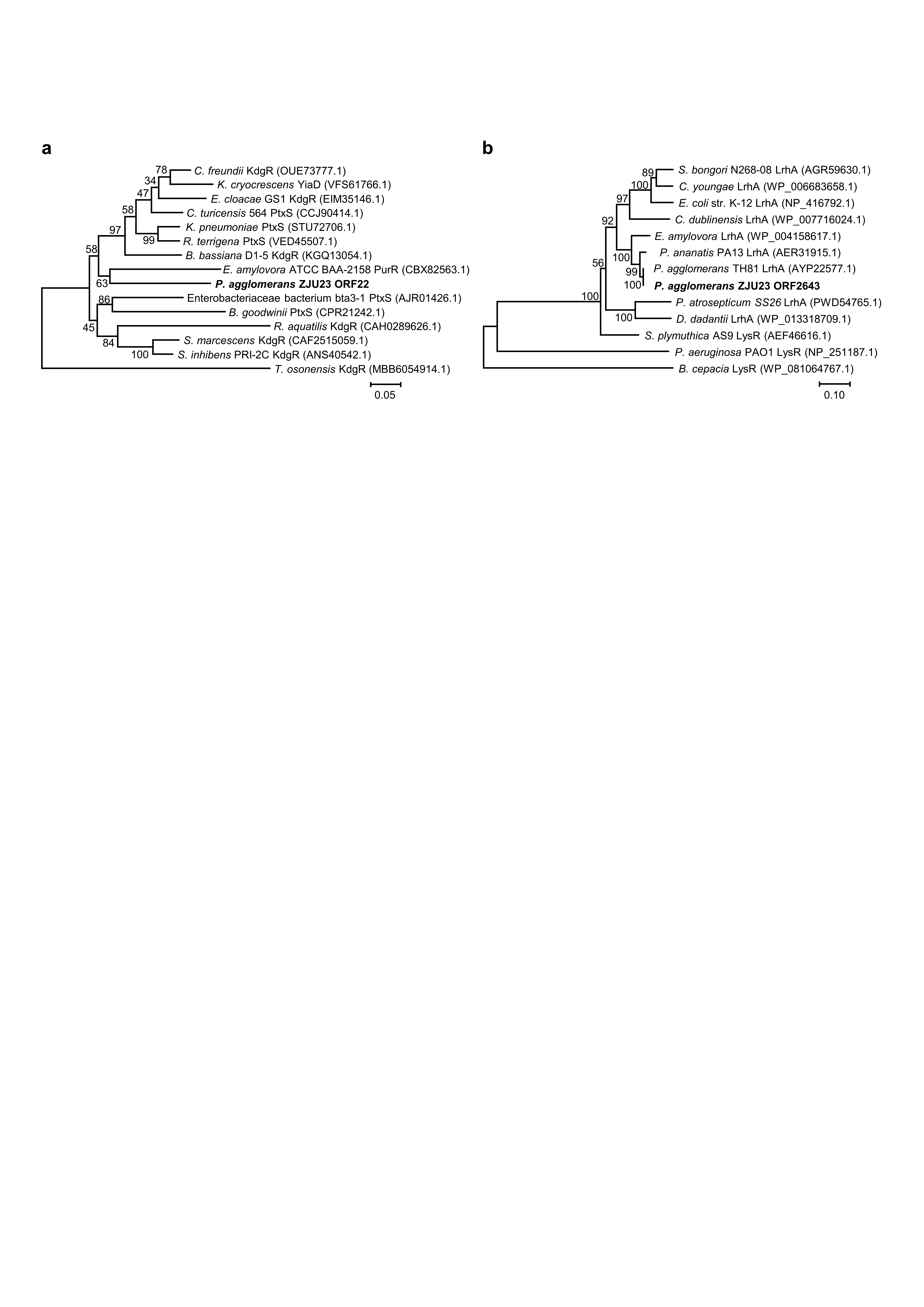

Supplement: Supplementary file 4 — Additional file 4: Fig. S3. Phylogenetic analysis based on the amino acid sequences of proteins encoded by ORF22 a and ORF2643 b in P. agglomerans ZJU23. [file 12934_2023_2051_MOESM4_ESM.tif]
